# Sexual and reproductive health needs of young people living with HIV in low- and middle-income countries: a scoping review

**DOI:** 10.1186/s12978-021-01269-7

**Published:** 2021-11-05

**Authors:** Laura S. Mkumba, Martha Nassali, Jasmine Benner, Tiarney D. Ritchwood

**Affiliations:** 1 Duke Global Health Institute, Durham, NC USA; 2 Department of Family Medicine and Community Health, Duke University, Durham, NC USA; 3 North Carolina Central University, Durham, NC USA

**Keywords:** HIV, Young people, Sexual and reproductive health, LMICs

## Abstract

**Background:**

Young people living with HIV(YPLWH) in low-and middle-income countries are entering adolescence and young adulthood in significant numbers. The majority of the HIV-related research on these young people has focused on clinical outcomes with less emphasis on their sexual and reproductive health (SRH). There is an increasing awareness of the importance of understanding and addressing their SRH needs, as many are at elevated risk of transmitting HIV to their sexual partners and young women, in particular, are at significant risk for transmitting HIV to their infants. The purpose of this scoping review is to synthesize research investigating the SRH needs of young people living with HIV in low- and middle-income countries.

**Methods:**

We searched electronic databases for studies focusing on young people aged 10–24 years and 27 studies met inclusion criteria.

**Results:**

This review identified four themes characterizing research on SRH among young people living with HIV: knowledge of SRH, access to SRH services, sexual practices, and future family planning and childrearing.

**Conclusions:**

Our findings suggest a need for additional research on comprehensive sexuality education to equip YPLWH with knowledge to facilitate desirable SRH outcomes, interventions on sero-status disclosure and condom use, and health provider capacity to provide SRH services in their pre-existing HIV clinical care.

## Plain Language summary

Young people living with HIV(YPLWH) in low-and middle-income countries are entering adolescence and young adulthood in large numbers. The majority of the HIV-related research on these young people has focused on clinical outcomes with less emphasis on their sexual and reproductive health. It is important to understand and address their sexual and reproductive health (SRH) needs, as many are at a high risk of passing on HIV to their sexual partners and young women, in particular, are at significant risk for passing on HIV to their infants. The purpose of this scoping review is to summarize research examining the SRH needs of young people living with HIV in low- and middle-income countries. We searched online databases for studies focusing on young aged 10–24 years and 27 studies were included in the review. This review identified four themes characterizing research on SRH among young people living with HIV: knowledge of SRH, access to SRH services, sexual practices, and future family planning and childrearing. Our findings suggest a need for additional research on comprehensive sexuality education to equip YPLWH with knowledge to facilitate desirable SRH outcomes, interventions on sero-status disclosure and condom use, and health provider capacity to provide SRH services in their pre-existing HIV clinical care.

## Background

The widespread success of highly active antiretroviral therapy (HAART) has improved the outlook and life expectancy of the nearly 3.4 million young people living with HIV worldwide [1]. While the majority of these young people (70%) were perinatally infected with HIV, the number of young people with behaviorally acquired HIV infection has increased overtime; in 2019, youth (aged 15–24 years) accounted for 28% of all new HIV infections, with adolescent girls and young women comprising up to two-thirds of new infections [1, 2]. HIV infection during adolescence and early adulthood poses a number of challenges to young people’s social and emotional development. Many young people living with HIV (YPLWH), for example, report difficulty establishing romantic relationships, experiences of stigma and discrimination, and fear the involuntary disclosure of their HIV status [3].

Considering that adolescence is characterized by a strong desire for autonomy and a rise in sexual expression and exploration [4], many YPLWH, like their peers without HIV, initiate sexual activity during this stage. Unfortunately, young people tend to have both low levels of sexual health knowledge and limited access to sexual and reproductive health (SRH) services, which are linked to higher engagement in sexual risk behaviors, unplanned pregnancies, and higher rates of sexually transmitted infections (STIs) [5, 6]. While these outcomes are concerning for all young people, the consequences are far more concerning for YPLWH, as they are at risk for transmitting the virus to their sexual partners and for young women, their infants, and experiencing worse health outcomes due to STI co-infection [7].

Though SRH has been identified as a research and health priority for young people, globally, many countries, including low and middle income countries (LMICs), have difficulty promoting SRH outcomes among young people [8, 9]. Structural and social barriers such as poverty, limited access to health services, and age of consent laws for accessing contraception and SRH services contribute to negative outcomes for young people in LMICs [10, 11]. For example, in comparison to their adult counterparts, young women from LMICs are at a greater risk for early pregnancy, unsafe abortions, and complications due to childbirth [12, 13]. YPLWH face an added burden of living with a stigmatized chronic condition that could impact their ability to access SRH services. Given the SRH challenges already impacting young people in LMICs, it is essential to understand how these challenges manifest in YPLWH in these countries. Understanding the unique SRH needs and identifying barriers to obtaining and accessing SRH services for YPLWH is necessary to support the development of comprehensive, youth friendly SRH interventions and services for this population. However, to date, we have limited research synthesizing the SRH needs of YPLWH from LMICs [10]. Published reviews have either focused on SRH needs of young people globally or SRH needs of young people in specific regions [8, 12, 14]. Therefore, in this article, we synthesize literature on the SRH needs of YPLWH in LMICs.

## Methods

We followed the Preferred Reporting Items for Systematic Reviews and Meta-Analyses Guidelines (PRISMA). An electronic search was conducted in March 2020 of the following databases: PubMed, Scopus, and Web of Science. Search terms included ‘adolescent’, ‘youth’, ‘HIV’, ‘AIDS’ ‘sexual and reproductive health’, ‘sexual behavior’, ‘reproductive health’, ‘family planning’, and ‘maternal and child health’. Eligibility criteria included the following: (1) published in a peer-reviewed journal; (2) written in English; (3) focused on YPLWH aged 10–24 years; (4) reported results from a LMIC; and (5) explicitly discussed the sexual and reproductive health of YPLWH. We manually identified countries as LMIC based on the World Bank classifications in February 2020.

All three databases were searched by three independent reviewers (LM, MN, and JB). Titles and abstracts were read and evaluated based on the above inclusion criteria. Articles that met inclusion criteria based on the first review were included in a spreadsheet and uploaded to a shared reference manager. Articles that were identified by at least two independent reviewers automatically moved to the full text review. Articles were included in this scoping review if all three reviewers agreed on its inclusion. Discrepancies amongst reviewers were discussed with the research group until an agreement was achieved. This scoping review focused on primary sources; however, other types of reviews (e.g., systematic, scoping, or content reviews) that were identified during article extraction were reviewed manually for any mention of articles that did not appear in our electronic search.

Data from articles were abstracted and charted detailing the author(s), year of publication, country setting, aims and purpose of the research, methodology, study population, and key findings. A qualitative content analysis was conducted on the key findings to identify common themes across studies.

## Results

**Fig. 1:**
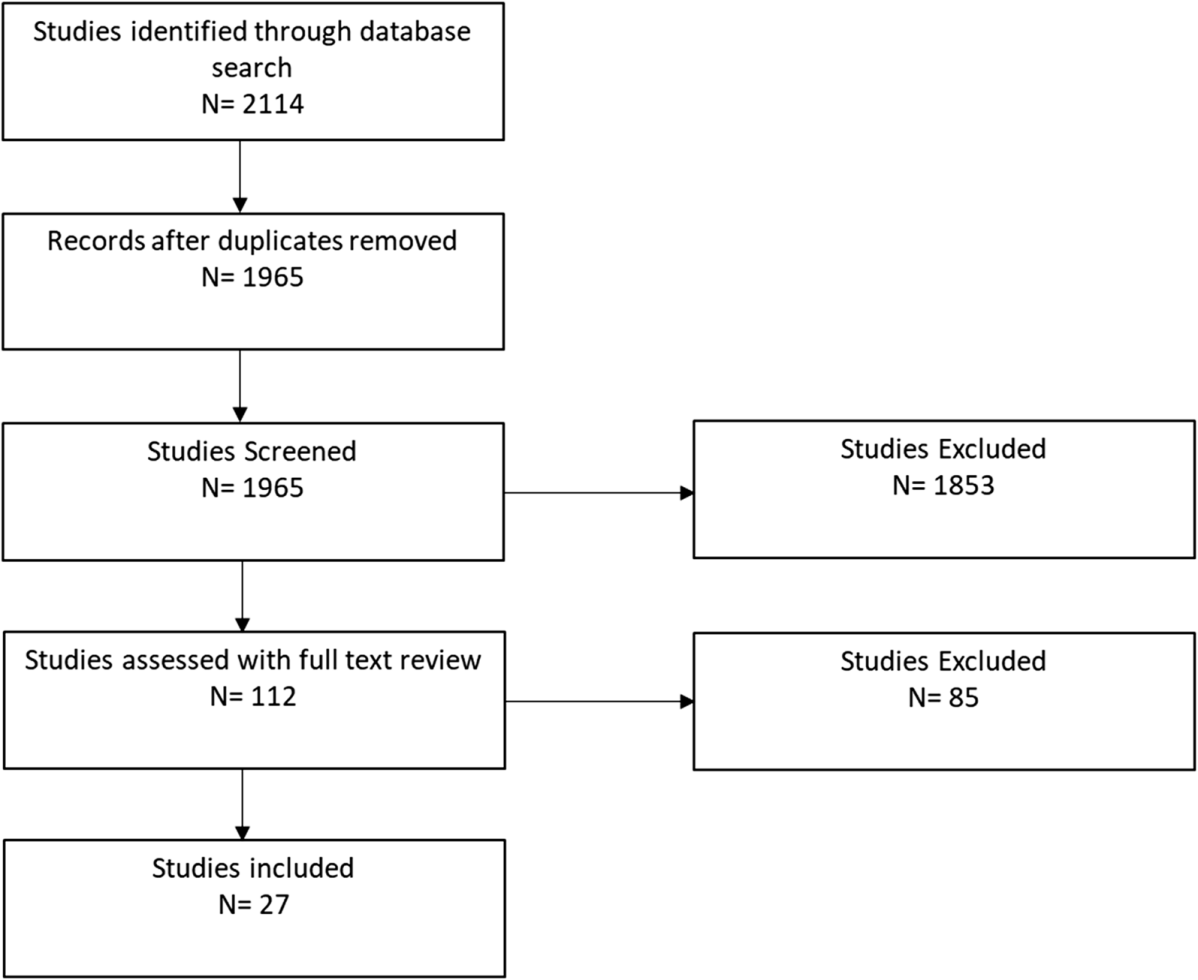
Flow diagram of study selection

Figure 1 presents the results of our study selection process. The initial search generated a total of 2114 titles and abstracts. After excluding duplicates and incomplete references, 1965 articles underwent title and abstract screening. Of these, 112 articles underwent full text review, and 85 articles did not meet inclusion criteria. The final sample consisted of 27 articles.

### Sample characteristics

Table 1 describes the characteristics of included studies, which were published between 2004 and 2019. Twenty-two studies were conducted in sub-Saharan African countries: Uganda (n = 7), Zambia (n = 5), Kenya (n = 3), Tanzania (n = 2), South Africa (n = 2), Côte d’Ivoire (n = 2), Malawi (n = 1), and Zimbabwe (n = 1). The remaining five studies were conducted in Asian countries: China (n = 1), India (n = 1), and Thailand (n = 3). Twenty-three studies had a cross-sectional study design and 11 studies utilized qualitative methods.

**Table 1:** Summary of Aims, Methods, Participants, and Key Findings of Studies Included in the Review

Authors	Year of publication	Country	Aims/purpose	Methodology	Study population	Key findings
Ankunda et al.	2016	Uganda	Describe sexual risk behaviors and factors associated with abstinence among YLWH in Uganda	Cross-sectional	338 youth Mean age: 19	Many participants were abstaining from sexual intercourse. Only a third of youth in relationships had disclosed their status to their partners. Less than half of the youth reported condom use in their last sexual encounter. 50% of participants reported preferring a HIV-negative partner. Tailored interventions that address disclosure and consistent condom use are needed to prevent HIV transmission
Arikawa et al.	2016	Côte d’Ivoire	Measure the incidence of pregnancy and its associated factors in ALWH	Retrospective Analysis	266 female adolescents Mean age: 12.8 years	Median age at first pregnancy was 17.7 years. All of the adolescents did not intend or plan to be pregnant, and the incidence of pregnancy was almost the same as adult cohorts in sub-Saharan Africa. There is an urgent need to provider adolescent-friendly reproductive health services
Bakeera-Kitaka et al.	2008	Uganda	Assess sexual and reproductive health needs and sexual risk taking among young people living with HIV	Qualitative Study: Focus group discussions	75 adolescents Mean age: 16 years	Adolescents had misconceptions about transmission and sexual and reproductive health. Fear of unintended consequences and hope for the future were the motivations for using protection during sexual encounters. Stigma, poverty, peer pressure, and ignorance of partners were some of barriers to using preventative measures. The study highlights the need for age-appropriate interventions to help youth adopt safe sexual habits
Birungi et al.	2009	Uganda	Understand the sexual and reproductive health needs of perinatally infected YLWH	Mixed methods: survey and in-depth interviews	732 adolescents Mean age: 17 years	Many adolescents were sexually active and desired to be in relationships. Majority (60%) of the youth who desired to be with HIV-negative partners wanted to avoid HIV re-infection. Only a third of those in relationships disclosed their status to their partner, and only one-third reported always using condoms
Birungi et al.	2011	Kenya	Examine the use of maternal health services among HIV-positive female adolescents	Cross-sectional: quantitative surveys	359 adolescent girls Age range: 15–19	Use of PMTCT services was lower than use of pre-natal services among HIV-positive adolescents. There was low utilization of skilled attendance in post-natal care for pregnancies that ended in miscarriage, abortion, or stillbirth. Pregnant YLWH need maternal services that integrate PMTCT and there is a need to implement PMTCT services tailored to HIV-positive adolescent mothers
Busza et al.	2013	Tanzania	Examine how adolescent experience their sexuality within the context of a home-based care program	Qualitative	14 adolescents (age 15–19) 10 caregivers 12 home-based care providers	Adolescents did not feel comfortable discussing sex and sexuality with their providers and caregivers. They also reported being discouraged from sexual activity. Adolescents expressed concerns with disclosure and infecting others and reported limited access to information on reproductive health. Caregivers and providers reinforced negative views ALWH engaging in sexual activity
Dago- Akribi et al.	2004	Côte d'Ivoire	Describe the psychosexual development of youth enrolled in the Yopougon Child Program	Qualitative	19 youth (age 13–17 years)	Youth were concerned with bodily development, getting married, and having children. There is a need for programs that support youth in their sexual development
Hodgson et al.	2012	Zambia	Explore and document the informational, psychosocial, sexual and reproductive health (SRH) needs of adolescents (aged 10–19 years) living with HIV in Zambia, and identify gaps between these needs and existing services	Qualitative: semi-structured interviews and focus group discussions	111 adolescents (10–19 years) 59 key informants (health care workers n = 38 and parents/guardians n = 21)	Social networks have significant impact on treatment adherence and assist adolescents in coming to terms with an HIV diagnosis. Service providers do not adequately meet the adolescents’ needs for SRH information Adolescents living with HIV require effective, targeted and sustainable HIV services to navigate safely through adolescence
Horwood et al.	2013	South Africa	Compare the characteristics of adolescent mothers and adult mothers, including HIV prevalence and MTCT rates	Quantitative: questionnaires	4485 adolescent mothers (12–19 years 14,608 adult mothers (20–39 years) 7800 infants (< = 16 weeks)	Despite high levels of antenatal clinic attendance among pregnant adolescents in KwaZulu-Natal, the MTCT risk is higher among infants of HIV-infected adolescent mothers compared to adult mothers. Adolescent mothers were less likely to receive the recommended PMTCT regimen. Access to adolescent-friendly family planning and PMTCT services should be prioritized for this vulnerable group
Landolt et al.	2017	Thailand	Assess strategies to improve safe-sex practices in sexually active female adolescents living with HIV, through linking reproductive health (RH) care with HIV care	Single arm prospective study	77 sexually active young women (12 – 24 years) Median age: 19 years	At baseline, two-thirds had disclosed their sero-status to their partner, and 68% reported condom use during the first intercourse/ Majority of participants showed improvement in knowledge of safe sex practices at post follow-up visits. Individual counseling was often rated as the most helpful source of information. Offering continuous reproductive care linked with HIV care resulted in increased uptake of hormonal contraception for dual protection
Lolekha et al.	2015	Thailand	Assessed the knowledge, attitudes, and practices of perinatally HIV-infected youth and youth reporting sexual risk behaviors receiving care at two tertiary care hospitals in Bangkok, Thailand	Cross-sectional: audio self-assisted interview	197 adolescents (11–18 years) Median age: 14 years	Most youth could correctly answer questions about HIV transmission and ARV adherence, but more than half lacked knowledge on family planning, STI health, and reproductive health Low condom use was reported in sexually active youth. The study highlighted the need for interventions and resources to improve knowledge on reproductive health and reduce engagement in risky behavior among HIV-infected youth in Thailand
Loos et al.	2013	Kenya Uganda	Assess the impact of HIV and related contextual conditions on identity formation of adolescents living with HIV/AIDS (ALH) in the domains of physical, cognitive, social, and sexual development	Qualitative: focus group discussions	119 adolescents (10–19 years) 54 care givers 55 service providers	Youth experimented with their sexuality as a means to discover their social identity. For many youths, sex served as a way of becoming “special” and coping with emotional pain of living with HIV
Mbalinda et al.	2015	Uganda	Explore the correlates of ever had sex among perinatally HIV-infected (PHIV) adolescents	Cross-sectional: survey	624 adolescents (10–19 years) Mean age: 16.2 years old	About three-fourths of participants reported not using condoms consistently and almost half did not use condoms in their last sexual encounter. Almost 50% of participants did not know the HIV status of their partners. Risk reduction interventions are required to minimize unplanned pregnancies, STI, and HIV transmission
Mburu et al.	2013	Zambia	Document psychosocial and sexual reproductive health care needs of ALHIV and identify gaps between those needs and currently available services for adolescents	Qualitative: in-depth interviews	111 adolescents 21 parents 38 health providers	ALHIV wanted greater access to information about HIV, SRH, and protective measures. They wanted services that offer privacy and confidentiality, short wait times and youth-friendly., Parents and caregivers agreed that SRH services could be improved. Interventions are needed to prepare adolescents for disclosure
McCarraher et al.	2018	Zambia	Advance our understanding of the reproductive health needs of ALHIV and to assess the extent to which these needs are being met	Mixed Methods: surveys and in-depth interviews	Surveys: 312 adolescents IDI: 32 adolescents 23 care givers 10 clinic staff	A fifth of participants reported having ever had sex and all desired to have children in the future. Adolescents reported low rates of disclosure. While adolescents knew about condom use, only half reported condom use at the last sexual encounter. Caregivers and service providers preferred to promote abstinence first followed by condom use. Half of the surveyed providers and caregivers supported offering contraceptive counseling at the ART clinic
Mu et al.	2015	China	Assess SRH and HIV knowledge and perceptions among perinatally HIV-infected adolescents	Cross-sectional: survey	124 adolescents (11–19 years) Median age: 15.6	Only 5% correctly answered all questions regarding HIV knowledge and pregnancy 79% of participants had never discussed puberty development or sexuality with parents, and less than 50% had ever heard of condoms. A fifth of the participants did not know how to get information on SRH and HIV
Mwalabu et al.	2017	Malawi	Explore the sex and relationship experiences of young women growing up with perinatally-acquired HIV in order to understand how to improve SRH care and associated outcomes	Qualitative- in-depth interviews	42 participants 14 cases Each case- young woman (15–19 years), caregiver, service provider	Young women reported wanting to be normal and seeking romantic relationships to find love and acceptance. Caregivers and service providers wanted young women to practice abstinence. Young women living with HIV need more support from healthcare providers and caregivers on reproductive health
Ndongmo et al.	2017	Zambia	Explore the sexuality and SRH experience and needs of adolescents living with HIV	Cross-sectional; mixed methods	148 adolescents (15–19 years) Mean age: 17 years	Majority of adolescents had sexual experience and expressed sexual desires and needs. Less than a fifth of participants had ever sought reproductive health services, and about half reported being able to discuss sexual issues with parents and caregivers. Over half had not disclosed their status to their sexual partners. Not being in school was a significant predictor, for knowing where to access information about sex
Ngilangwa et al.	2016	Tanzania	Inform SRH programs and identify best approaches to reach marginalized youth	Cross-sectional mixed methods	396 young people (10–24 years old)	64% of YPLWH had knowledge of existing SRH policies, and less than half reported talking to their parents about various SRH topics. Peer educators were the leading source of information followed by parents and teachers. Young people value multiple sources of information
Obare et al.	2010	Uganda	Explore policies for SRH in adolescents and identify barriers for SRH programming for adolescents	Qualitative: unstructured interviews	23 key informants from bilateral institutions, civil society organizations, and NGO	While there are broad policies to address SRH in adolescents, there is a gap in policies that address SRH for ALWH. There is also a need to increase capacity of HIV providers to offer SRH services to ALWH
Obare et al.	2012	Kenya	Examine factors associated with unintended pregnancies, poor birth outcomes and post-partum contraceptive use in Kenyan ALWH	Cross-Sectional Structured interviews	394 adolescent girls (age 15–19) who had ever been pregnant	Girls who had begun childbearing had experienced multiple unintended pregnancies. There has been a lack of training for providers to counsel ALWH on SRH also existing programs focus on abstinence-based education. Poor birth outcomes were less likely in cases where the pregnancy occurred within a marital union. Post-partum contraceptive use was inconsistent
Okawa et al.	2018	Zambia	Assess the sexual behaviors and SRH needs of ALWH in Zambia and identify any concerns with marriage and desire to have children	Cross-Sectional Self-administered structured questionnaire	175 adolescents (15–19 years old)	One-third of youth did not use a condom during first intercourse. Most of the adolescents desired to have children, and about half were concerned about intimate relationships, disclosing their status and potential rejection after disclosure. Adolescents had an unmet need for SRH services and intimate relationships
Rolland-Guillard et al.	2019	Thailand	Compare risky sexual behavior, planning for the future, and reproductive health between perinatally-infected youth and non-infected controls	Case control study, self-administered questionnaires	571 adolescents (12–19 years old)	Sexual habits did not differ between PHIVA and their counterparts, but PHIVA tended to have less expectations for education and family formation. PHIVA experienced puberty later than adolescents in the general population. Need for psychosocial services and policies in place to foster future planning for PHIVA
Vranda et al.	2018	India	Explore the SRH needs and concerns of ALWH in India	Qualitative in-depth interviews	20 adolescents (13–18 years old)	Youth were worried about finding romantic partners and having children. Most youth had not discussed SRH with service providers, parents, guardians. There is a lack of adolescent-friendly services in India
Vu et al.	2017	Uganda	Examine the effectiveness of the Link up Project- a peer led intervention that provided comprehensive care package of HIV and SRH services to Ugandan YLWH	Pre-post cohort study with 9-month follow-up Quantitative measures and IDIs	473 youth (15–24 years old)	Linkage services increased uptake of HIV and SRH clinical services in the study population. At endline, youth demonstrated increased comprehensive knowledge on HIV transmission and other SRH topics. Youth also reported increase comfort and self-efficacy to discuss SRH topics with their providers
Vujovic et al.	2014	South Africa	To describe the SRH opinions and concerns of very young adolescents in South Africa	Qualitative: In-depth interviews and FGD	27 adolescents (10–14 years old) 9 healthcare providers	Adolescents knew little about their bodies but wanted to learn more about sexual and reproductive health issues. Healthcare providers were not confident about providing SRH services to ALWH and highlighted the need for capacity building health service staff. The study identified a need to address sexual and reproductive health in early adolescence and make sexual and reproductive health an integral part of youth health services
Zamudio-Hass et al.	2012	Zimbabwe	Understand how young women living with HIV weigh disclosure in the context of forming partnerships and childbearing	Qualitative: in-depth Interviews	28 young women (16–20 years old)	Disclosure played a big role in deciding childbearing or birth spacing because it was a turning point in romantic relationships. Women reported support and acceptance and abuse and rejection when they disclosed their HIV status to their partners. Women wanted healthcare providers to do more in helping them navigate disclosure

Our review identified four themes characterizing SRH needs among YPLWH: knowledge of SRH, access to SRH services, sexual practices, and future family planning and childrearing. While the resulting studies were conducted in different countries and communities, the themes were consistent across geographical regions. We describe each theme in detail below.

### Knowledge of sexual and reproductive health

Thirteen studies reported that YPLWH either had low SRH knowledge or limited access to information about SRH. Five studies showed that YPLWH lacked an understanding of how HIV affected their bodies, ways in which HIV and other STIs were transmitted, or had limited SRH knowledge [7, 15–18]. A study of YPLWH with perinatal HIV infection in China, for example, found that only 5% of enrolled young people were able to correctly answer questions about HIV, and only 18% of young people answered questions about contraception correctly [16]. In contrast, a knowledge, attitudes, and practices study in Thailand found that, while most young people could correctly answer questions about HIV transmission and antiretroviral therapy adherence, fewer than half demonstrated knowledge on family planning, reproductive health, and STIs [19].

Additionally, YPLWH reported that they did not know where to go to obtain information about SRH such as contraception [16, 20]. In contrast, YPLWH who did know where to obtain information reported discomfort discussing SRH with providers and sometimes avoided seeking services due to fear of being stigmatized or judged [21]. In a qualitative study in Tanzania, young people reported discomfort with discussing sex and sexuality with providers and caregivers, and also had limited access to information on reproductive health [22].

### Access to reproductive health services

Four studies assessed maternal health and pregnancy among adolescent girls living with HIV [23–26]. These studies found that, while the incidence of unplanned pregnancies among adolescent girls living with HIV was similar to the incidence in adult cohorts in sub-Saharan Africa, utilization of prenatal care and participation in prevention of mother-to-child transmission (PMTCT) health services was lower among adolescent girls living with HIV [24]. These finding may partially explain the results of a study in South Africa suggesting that YPLWH have a higher likelihood of vertically transmitting HIV to their babies than their adult counterparts [25]. In addition to low engagement in prenatal care and PMTCT services, YPLWH in Côte d’Ivoire and Kenya, for example, also reported inconsistent post-partum contraception use, which has been linked to higher rates of unintended pregnancies within this population [23, 26].

Provider characteristics were identified as barriers to accessing SRH services among YPLWH. We identified nine studies of healthcare providers and other health service delivery stakeholders that assessed their comfort, readiness, and/or competence to provide SRH to YPLWH. In four studies, providers reported low self-efficacy in their ability to provide SRH services such as contraception to YPLWH [18, 22, 27, 28]. Common reasons for low confidence were cultural barriers, gaps in knowledge about SRH needs of YPLWH, and having a limited number of healthcare providers available to offer comprehensive services. Other healthcare provider-related barriers included personal and often value-laden preferences for abstinence among young people, with some suggesting condom use as an alternative option for those were unable to abstain from sexual activity [21, 29]. Our review also identified the absence of clear policies to guide SRH service provision for YPLWH. A Ugandan study of key stakeholders from non-governmental organizations and bilateral organizations, for example, linked gaps in SRH policies for YPLWH to the absence of youth-friendly healthcare transition clinics and low provider and institutional capacity to provide these services [30].

### Sexual practices

Collectively, findings from reviewed studies suggested that YPLWH engaged in sexual activities at rates that were similar to their peers without HIV [31]. Moreover, like their peers without HIV, YPLWH reported inconsistent condom use, with fewer than half reporting condom use during their last sexual encounter [32–35]. While the causes of inconsistent condom use within this population are multifactorial, a Ugandan study suggested that the fear of unintended consequences, such as pregnancy and contracting other STIs, motivated YPLWH to use condoms [15].

YPLWH also had the added burden of disclosing their HIV status to their sexual partners, which was often accompanied with fear of HIV-related stigma and rejection by sexual partners [22, 32]. Our review revealed that more than half of YPLWH reported low levels of HIV status disclosure to their sexual partners. Moreover, many of these young people were unaware of the HIV serostatus of their sexual partners [19, 20, 29, 32–36]. Two Ugandan studies of YPLWH reporting young people’s preferences for an HIV-negative partner due to fear of HIV re-infection [32, 33].

### Future relationships and childbearing

Lastly, in four studies, concerns about future relationships and childbearing emerged as an SRH theme for YPLWH in LMICs. YPLWH reported desires to have children and be married in the future, but also expressed concerns with realizing these desires [35, 37, 38]. A Thai study, for example, suggested that YPLWH had lower expectations about family formation than their peers without HIV [39]. For many young people, these feelings were partially a result of messaging from caregivers and healthcare providers on the dangers of sexual intercourse and the importance of abstinence [35]. Additionally, YPLWH were less hopeful of future partnering due to the fear of disclosing their status to their partners and experiencing rejection and abandonment. This fear was often coupled with uncertainty about how to disclose their status to future intimate partners. Young people also expressed concerns about transmitting the virus to their sexual partners and children and reported low knowledge regarding how to prevent sexual transmission to others [35].

## Discussion

This scoping review synthesized literature elucidating the sexual and reproductive health needs of YPLWH in LMICs. The review yielded 27 studies from 11 countries in sub-Saharan Africa and Asia. We identified four main sexual and reproductive health themes: knowledge of sexual and reproductive health, access to sexual and reproductive health services, sexual practices, and future relationships and childbearing. The findings from this review highlight specific gaps to be addressed in future research, policy, and programs in LMICs. First, there is a need for youth-friendly, sexual and reproductive health services for YPLWH in LMICs that integrate comprehensive sexuality education, which is characterized by open-mindedness, non-judgmental and positive attitudes by health providers. Second, there is a need for interventions that encourage young people to adopt safe sex behaviors, such as condom use, and prepare young people to disclose their HIV status to sexual partners.

Inadequate knowledge of sexual and reproductive health, contraceptive options, and family planning coupled with limited access to sexual reproductive health services was a common finding across the studies. This finding has important implications for the health outcomes of YPLWH, particularly as it relates to SRH for adolescent girls living with HIV. Generally, adolescent girls and young women in LMICs have an increased risk of acquiring HIV and having unintended pregnancies, unsafe abortions, and birth complications [8]. These risks are compounded for adolescent girls and young women living with HIV, as they are at increased risk of spontaneous abortions, pre-term delivery and delivering babies of low birth weight [40]. Additionally, these findings have implications for reducing mother to child transmission of HIV. Despite scale up of PMTCT services globally, rates of mother to child transmission have declined modestly in many LMICs (UNAIDS). In order to achieve gains in PMTCT, YPLWH and especially adolescent girls and young women living with HIV must receive comprehensive sexual and reproductive health knowledge and services.

Our findings showed that YPLWH engage in sexual encounters at similar rates to young people not living with HIV, and YPLWH desire to be in relationships and have families. Interestingly, in two studies YPLWH reported a preference for romantic and/or sexual partners without HIV [32, 33]. This is potentially problematic, as HIV status disclosure to sexual partners was relatively low among YPLWH in the study cohorts and many were unaware of their partner’s HIV status [20, 33, 34]. YPLWH have a right to desire partnership with any person regardless of the person’s sero-status; however, low rates of disclosure and inconsistent condom use puts their partners and themselves at risk for HIV reinfection, STI co-infection, and HIV transmission. HIV sero-status disclosure is a key component in reducing HIV transmission; however, fear of stigma, violence, and rejection pose as obstacles for YPLWH. These findings point to an urgent need to implement disclosure related interventions targeted towards YPLWH. These interventions should include education on consistent condom use,comprehensive counseling on disclosure and negotiating safe sex practices with partners, and the importance of maintaining an undetectable viral load to prevent HIV transmission [3].

Lastly, findings from this review highlight the gap in health provider capacity to provide SRH services to YPLWH. Providers preferred abstinence-based SRH education and counseling. However, given that YPLWH continue to be sexually active despite receiving abstinence-based counseling and messaging, this demonstrates that health providers are not responding adequately to the needs of YPLWH. Lack of training in adolescent psychosocial development and the unique SRH needs of YPLWH has been linked to poor quality of care and low motivation to engage in SRH services by YPLWH [18, 22, 27, 28]. These results support the need for clear guidelines on providing SRH services to YPLWH along with additional training for health providers to adequately respond to the needs of this population [28, 30]. These trainings should address providers’ attitudes and belies on young people’s sexuality, and sensitize providers to the needs and rights of young people.

This review indicated that there are a few published interventions aimed at improving SRH among YPLWH in LMICs [41]. We identified two interventions that integrated comprehensive sexual and reproductive health services with HIV care, which resulted in greater SRH knowledge among YPLWH and a significant increase in the uptake of SRH services [7, 42]. Providing youth-friendly services that are incorporated with HIV services is a promising method of improving knowledge and access to SRH for YPLWH in LMICs. Youth-friendly services, for example, may create an acceptable, equitable, and confidential environment for YPLWH to receive the SRH care that they require [43]. Youth-friendly services should also offer comprehensive sexuality education to equip YPLWH with adequate knowledge to make informed decisions. Comprehensive sexual education provides young people with information regarding anatomy but also empowers them to adopt healthy sexual habits [9]. As YPLWH transition into adulthood and explore their sexual and relationship desires, it is important to provide them with the resources and tools necessary to navigate aspects of their lives that are both directly and indirectly linked to their SRH, which will include coping skills necessary to live with a stigmatized chronic condition that impacts their romantic and sexual outlook, family planning, and preparation for disclosing their serostatus to intimate partners.

This study is not without limitations. First, we limited our search to the three most well-utilized databases in public health and HIV research. As such, it is possible that some studies may have been missed and not included in this review. Nonetheless, a scoping review is not meant to be a systematic assessment of the literature on a specific topic. Instead, they are best utilized when topics are constantly evolving, as is the case of SRH among YPLWH in LMICs. Second, results were limited to studies published in English and may have led to a language bias. Third, the studies focused on YPLWH in SSA countries and a few countries in Asia. As a result, it is possible that these findings may not be generalizable for YPLWH outside of these settings. Despite these limitations, the findings in this review add to the growing body of knowledge on the unique SRH challenges of YPLWH. To the best of our knowledge, this is the first scoping review that has documented the SRH needs of YPLWH in LMICs [44–46].

## Conclusion

As YPLWH begin to transition to adulthood, they face the compounded burden of navigating a stigmatized, chronic condition while also experiencing the rapid physiological and sexual development characteristic of adolescence and young adulthood. While sexual and reproductive health is a key component of this developmental stage, additional research was needed to synthesize the unique SRH needs of YPLWH in LMICs. Our findings suggest a need for additional research on comprehensive sexuality education to equip YPLWH with knowledge to facilitate desirable SRH outcomes, interventions on sero-status disclosure and condom use, and health provider capacity to provide SRH services in their pre-existing HIV clinical care.

## Data Availability

Data sharing is not applicable as no new datasets were generated or analyzed during the conduct of this review.

## Abbreviations

HAART: Highly active antiretroviral therapy
LMICs: Low and middle income countries
PMTCT: Prevention of mother-to-child transmission
PRISMA-c: Preferred Reporting Items for Systematic Reviews and Meta-Analyses
SRH: Sexual and reproductive health
STIs: Sexually transmitted infections
YPLWH: Young people living with HIV
